# Prevalence of C*ryptosporidium* spp. infection in a working horse population in Egypt

**DOI:** 10.1007/s11250-023-03773-3

**Published:** 2023-10-18

**Authors:** Shebl E. Salem, Amany M. Abd El-Ghany, Hussein A. Elsheikh, Enas M. Abdel-Ghany, Refaat Ras

**Affiliations:** 1 Department of Surgery, Faculty of Veterinary Medicine, Zagazig University, Zagazig, 44519 Egypt; 2 Department of Parasitology, Faculty of Veterinary Medicine, Zagazig University, Zagazig, 44519 Egypt; 3 The Veterinary Clinic, Faculty of Veterinary Medicine, Zagazig University, Zagazig, 44519 Egypt; 4 Genetic and Cytology Department, Biotechnology Research Institute, National Research Centre, Giza, Egypt; 5 Department of Microbiology and Parasitology, Faculty of Veterinary Medicine, Badr University in Cairo (BUC), Badr City, Cairo, Egypt

**Keywords:** Horse, Working equid, Egypt, *Cryptosporidium* ssp., Prevalence

## Abstract

Working horses support the livelihoods of smallholder farmers in Egypt. No previous study has investigated the prevalence of cryptosporidiosis in working horses in Egypt. Faecal samples were collected from 607 working horses recruited from thirty-seven villages/areas in two Egyptian governorates and examined for *Cryptosporidium* spp. infection using the modified Zielh-Neelsen staining technique. Data on signalment, history of recent diarrhoea, and strongyle burden were collected. The prevalence of *Cryptosporidium* spp. infection was calculated using a bootstrap method and potential risk factors for infection were investigated using mixed-effects logistic regression models that included sampling location as a random-effects variable. The prevalence of *Cryptosporidium* spp. infection was 28.7% (95% confidence interval = 23.5–33.9). None of the variables investigated, which include age, sex of the animals, and strongyle burden, were associated with risk of infection. This study provided evidence-based information on the prevalence of *Cryptosporidium* spp. infection in the study area. However, the potential zoonotic risk of *Cryptosporidium* cannot be confirmed until further studies are conducted to genotype these parasites.

## Introduction

Working horses play an important role in supporting agricultural work and the livelihoods of smallholder farmers in Egypt (Salem et al. 2017a, 2017b). *Cryptosporidium* spp. infection is a recognised cause of diarrhoea in immunocompetent foals as well as in foals with concurrent immunodeficiency (Bjorneby et al. 1991; Coleman et al. 1989). It has also been diagnosed in foals without clinical signs of diarrhoea (Netherwood et al. 1996; Veronesi et al. 2010). The prevalence estimates of *Cryptosporidium* spp. infections are variable. An earlier study reported a 100% prevalence in 22 foals, of which 14 foals (64%) had diarrhoea (Coleman et al. 1989). Prevalence in adult horses varied according to geographic location of the study and the detection method used; prevalence estimates reported ranged between 1.8 and 39% (Guo et al. 2014; Johnson et al. 1997; Li et al. 2019; Majewska et al. 2004; Veronesi et al. 2010; Xu et al. 2023).

Factors reported to be associated with shedding *Cryptosporidium* spp. oocysts in horses are history of diarrhoea, age < 6 months old, and breeding farm (Cole et al. 1998; Veronesi et al. 2010). Although molecular studies have indicated that humans and horses are infected with differing *Cryptosporidium* spp. subtypes, a few studies reported horse genotype *Cryptosporidium* infection of human subjects who had a history of direct contact with horses (Lebbad et al. 2021; Xiao et al. 2009; Zajaczkowska et al. 2022). A historical report of an outbreak of cryptosporidiosis in a veterinary hospital with multiple species involved which included humans also supports the zoonotic risk of the protozoan (Konkle et al. 1997). A more recent report from an equine perinatology unit described zoonotic transmission between foals and 6 students (Galuppi et al. 2016)

Studies that investigated *Cryptosporidium* spp. prevalence in animals in Egypt reported prevalences of 10.2% and 12.3% in cattle and buffalo, respectively (Ibrahim et al. 2016), and 34% in pet dogs (Gharieb et al. 2018). To the best of our knowledge, no previous studies in Egypt have investigated the prevalence of *Cryptosporidium* spp. in horses. Therefore, the objectives of the current study were to estimate the prevalence of *Cryptosporidium* spp. in working horses in Egypt and identify potential risk factors for *Cryptosporidium* spp. oocyst shedding.

## Material and methods

### Study population and recruitment

Faecal samples were collected as part of a cross-sectional study that investigated the prevalence of gastrointestinal nematode infection and anthelmintic resistance in working horses in two Egyptian governorates (Salem et al. 2021). Briefly, visits to the villages were arranged with the help of local veterinary surgeons. On each visit, a mobile clinic was used, and the villagers were informed about the presence of the clinic using a loudspeaker. We announced a free clinical and parasitological examination and a free anthelmintic treatment to encourage horse owners to bring their horses to the clinic. When it was deemed impractical for horse owners to bring their horses to the clinic, they were visited at their residences to collect samples. Sample size calculations to identify *a Cryptosporidium* spp. prevalence of 10% (Hatam-Nahavandi et al. 2019) with a precision of 5% and a 95% confidence interval (CI) indicated that recruitment of 138 horses was required. To account for clustering within villages, this initial sample size was multiplied by a design effect of 3.9 which was calculated assuming an intra-cluster correlation coefficient of 0.1 and that 30 horses would be sampled per village. This resulted in a total sample size of 538 horses (Dohoo et al. 2014).

### Sample and data collection

Faecal samples (about 200 g) were collected either from freshly voided faeces or manually from the rectum. Samples were placed in sealable plastic bags, with as much air as possible being expelled before sealing and were transferred to the laboratory where they were stored at 4 °C until processing. Information about horse signalment (age, sex, number of horses in the premises) was collected. Body condition score (BCS) was recorded on a scale of 1–9, where 1 indicated a poor BCS and 9 indicated an extremely fat BCS (Henneke et al. 1983). Sampling was conducted between December, 2019, and February, 2020.

### Parasitological examination

The level of strongyle burden was evaluated previously (Salem et al. 2021). *Cryptosporidium* spp. infection was diagnosed using direct stained smears. A faecal sample (approximately 5 g) was thoroughly mixed with 40 ml tap water and the mixture was then passed through a 250-μm-aperture sieve to remove debris. The mixture was then transferred to two 15-ml plastic conical tubes and allowed to settle in the refrigerator overnight. The supernatant was discarded, and a drop of the sediment was deposited on a microscope slide and spread using a cotton swab. The slide was left to dry at room temperature before staining using the modified Zielh-Neelsen staining technique (Henriksen and Pohlenz 1981). Each stained slide was examined using a light microscope under an oil immersion objective (× 100 magnification). The sizes of the identified *Cryptosporidium* spp. oocysts were measured using a micrometre eyepiece. Samples containing at least one 3–6 μm diameter, densely, or irregularly stained red, spherical body were considered positive (Ebrahimi Warkiani et al. 2011; Henriksen and Pohlenz 1981).

### Molecular analysis

Samples showed large number of oocysts microscopically were selected for further molecular analysis. DNA was extracted from 15 faecal samples using the QIAmp DNA Stool Mini Kit (Qiagen, Germany) following the manufacturer’s recommendations. Prior to extraction, faecal samples underwent five freeze-thaw cycles of freezing at – 20 °C for 10 min and thawing at 37 °C for 5 min. Extracted DNA was stored at – 20 °C until processing. Nested PCR assays for *Cryptosporidium* ssp. were performed by amplifying the 18S small subunit (SSU) rRNA gene, as previously described (Ryan et al. 2003). The forward primer 18SiCF2 (5′-GAC ATA TCA TTC AAG TTT CTG ACC-3′) and the reverse primer 18SiCR2 (5′-CTG AAG GAG TAA GGA ACA ACC-3′) were used in the primary PCR reaction. In the secondary reaction, forward 18SiCF1 (5′-CCT ATC AGC TTT AGA CGG TAG G-3′) and reverse 18SiCR1 (5′-TCT AAG AAT TTC ACC TCT GAC TG-3′) primers were used. The PCR reaction mixture (25 μl) consists of 12.5 μl Taq 2X Master Mix (New England, Biolabs), 1 μl of each primer (10 μM), 10.5 μl of PCR grade water, and 1 μl of extracted DNA. A two-step nested PCR protocol was used. The primary PCR reaction conditions consisted of an initial hot start at 94 °C for 5 min, followed by 40 PCR cycles of 94 °C for 30 s, 58 °C for 30 s, and 72°C for 30 s, then a final extension at 72 °C for 10 min. The second PCR reaction was performed using 1 μl from the primary PCR reaction product as a template DNA and the same PCR reaction conditions as the primary PCR. All secondary PCR products were examined with electrophoresis in 1.5% agarose in 1× TAE buffer gel stained with RedSafe^TM^ nucleic acid stain (Biovision, Egypt).

### Data analysis

Descriptive statistics were calculated for all variables (age, sex, BCS, strongyle infection [≥ 200 eggs per gram], duration of ownership, and number of horses kept by the same owner). The average within-village prevalence of *Cryptosporidium* spp. infection and the associated 95% CI was calculated following adjustment for clustering using a bootstrap method (Lesnoff and Lancelot 2012). A two-level random intercept logistic regression model that included sampling location as a random-effects variable was fitted to explore the association between explanatory variables and *Cryptosporidium* spp. infection. The model was fitted using the glmer::lme4 function (Bates et al. 2014) in R. Initially, the significance of the random-effects variable was examined by comparing null models (models without fixed-effects variables) with and without the random effect using a likelihood ratio test (LRT). Intraclass correlation coefficient (ICC), which measures the proportion of variance in log odds of *Cryptosporidium* spp. infection due to differences between locations, was calculated using the aod::iccbin function in R. The function uses a Monte Carlo simulation to calculate ICC (Lesnoff and Lancelot 2012). Age was included in the model as a linear fit based on the results of generalised additive models (Hastie 2015). None of the variables investigated was found to be significantly associated with *Cryptosporidium* spp. infection; therefore, a multivariable model was not built.

## Results

**Fig. 1:**
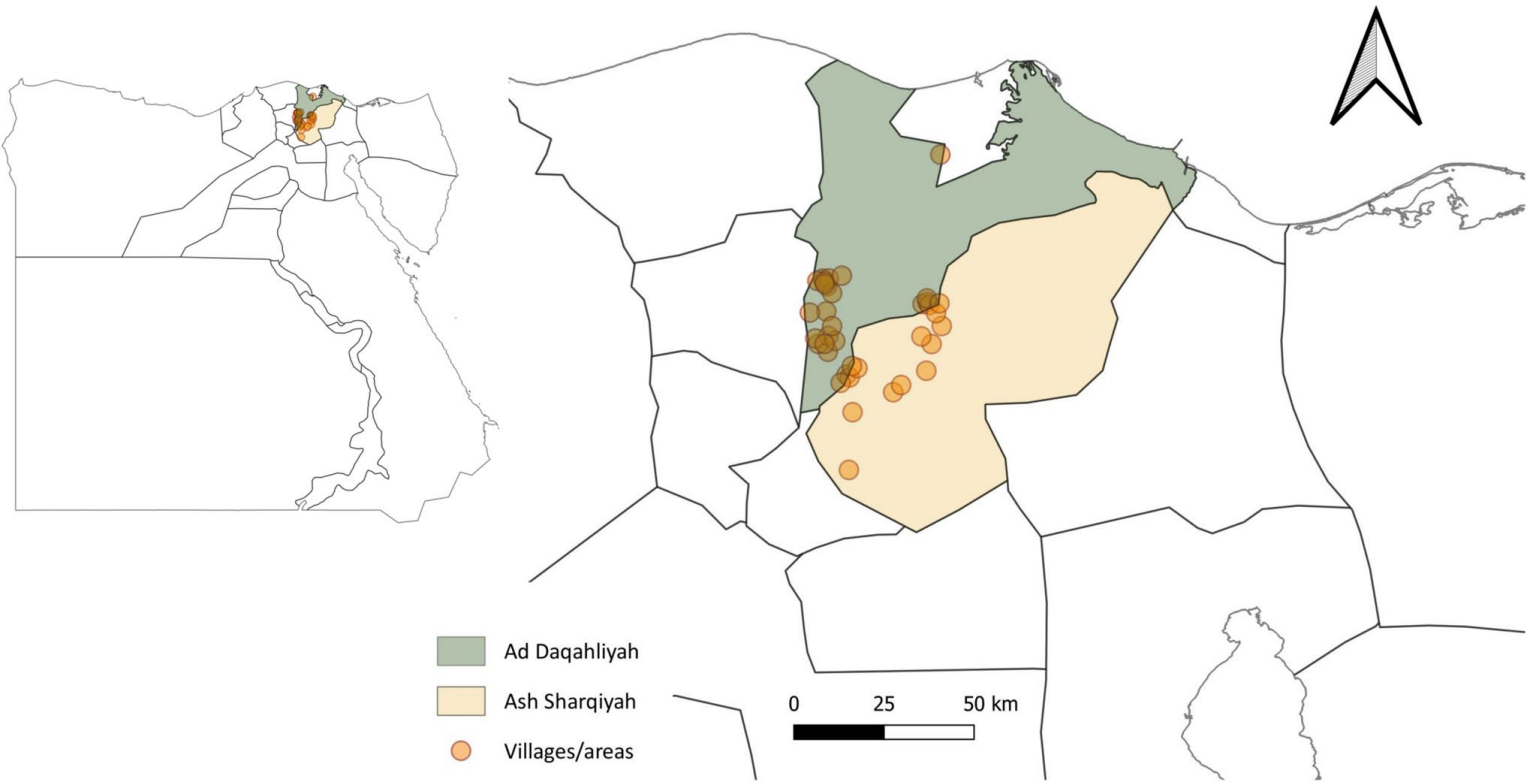
Map of locations from which horses were recruited into the study. The orange dots represent the approximate locations of the visited villages

Samples were collected from 37 villages/areas in the Al Dakahliya and Al Sharkia governorates which are situated in the northern part of Egypt approximately 100 km to the north of the capital Cairo (Fig. 1). The original study included 644 horses (Salem et al. 2021), but only 607 horses were examined for *Cryptosporidium* spp. infection in the current study. The horses were owned by 503 different owners and had a median age of 5 years (interquartile range [IQR] 2.5, 10) and included 473 females (77.9%) and 134 (22.1%) entire males. The horses had been under the present owner’s care for a median of 2.5 years (IQR 1, 5). The body condition score was recorded for 478 horses, with a median score of 4 (IQR 4, 5). All horses included in the study were apparently healthy without clinical signs or a recent history of diarrhoea.

**Fig. 2:**
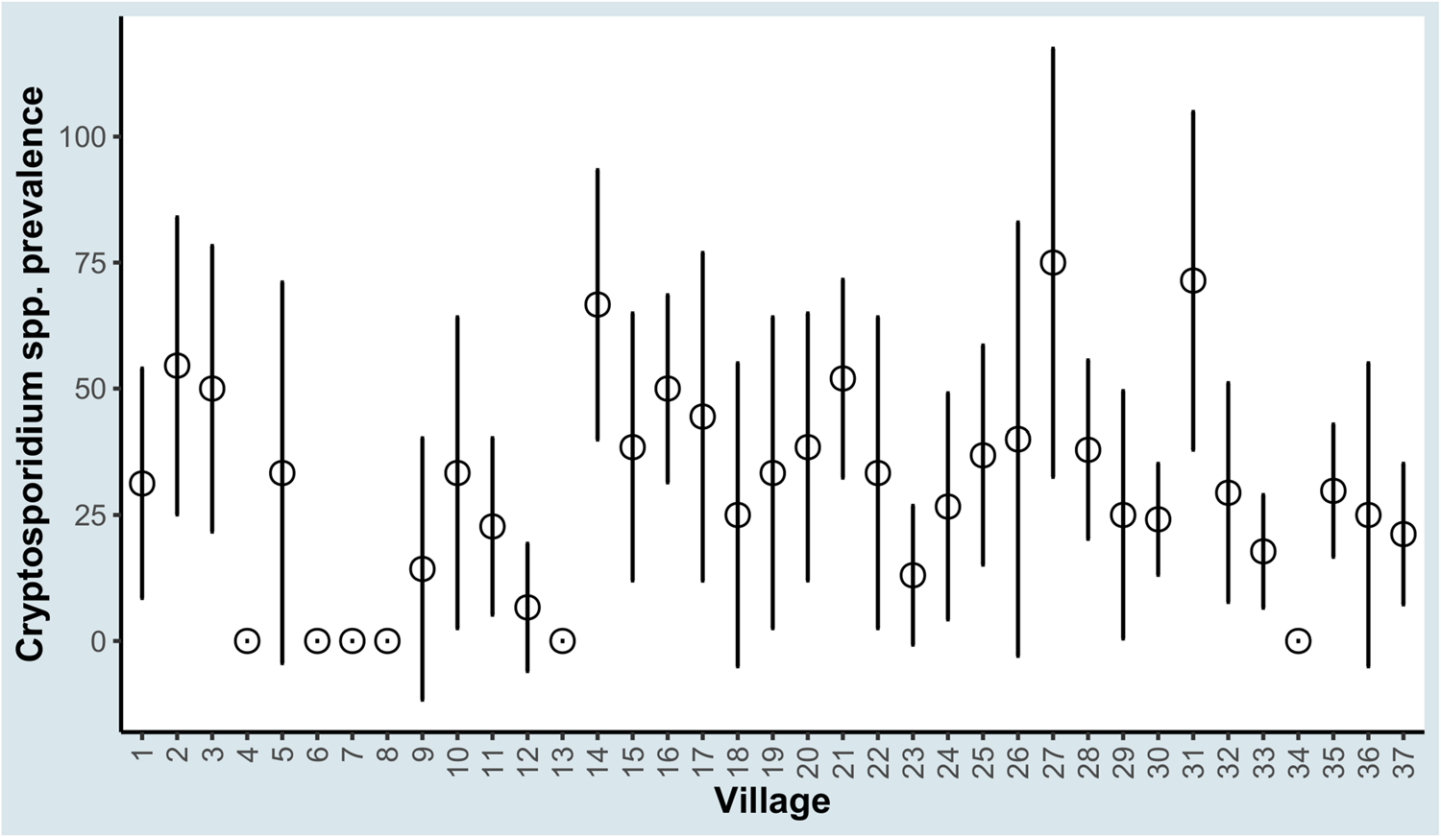
Prevalence and associated 95% Wald confidence intervals of *Cryptosporidium* spp. infection in 37 villages in Egypt. The circles represent prevalence, and bars represent the lower and upper 95% Wald confidence intervals

**Fig. 3:**
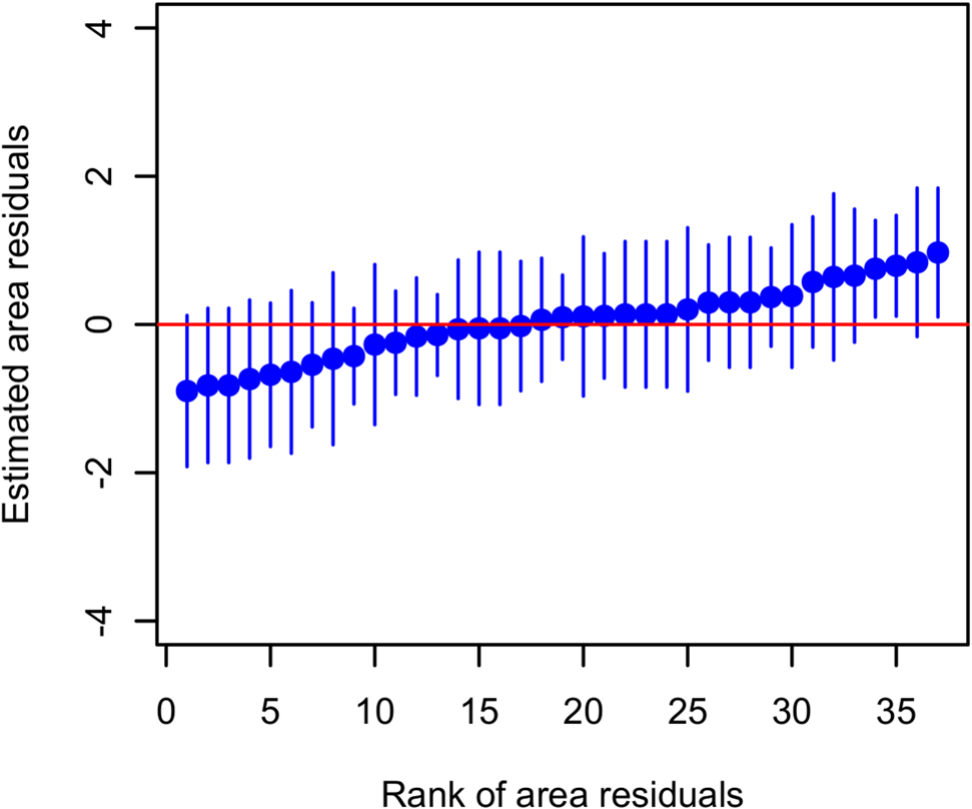
Caterpillar plot of the village/area random effect. The *y*-axis is the estimated residuals for sampling location, and *x*-axis is the rank of location residuals. The vertical lines represent the 95% CIs for the estimated residuals

The prevalence of *Cryptosporidium* spp. infection was 28.7% (95% CI = 23.5–33.9). *Cryptosporidium* spp. prevalence and the associated 95% Wald CI in each of the visited villages are presented in Fig. 2. The ICC of the village random effect was statistically significant (LRT *p* < 0.001) and indicated that approximately 8.5% of the residual variation in *Cryptosporidium* spp. infection was attributed to village characteristics. The village random effect was only statistically significant for three villages, whereas the remaining residuals were not different from the mean because the respective CIs included zero (Fig. 3). None of the PCR reactions performed yielded positive results.

Descriptive statistics and the results of univariable random-effects logistic regression models of categorical explanatory variables are presented in Table 1. None of the variables investigated were significantly associated with *Cryptosporidium* spp. infection. Male horses were at a numerically increased risk of being diagnosed with *Cryptosporidium* spp. infection, but the relationship did not reach statistical significance (*P* > 0.05). Age was evaluated as a linear fit in the logistic regression model and found to be non-significantly associated with *Cryptosporidium* spp. infection (OR = 0.98, 95% CI = 0.95–1.02, *P* = 0.5). BCS was also not associated with *Cryptosporidium* spp. infection (OR = 0.9, 95% CI = 0.8–1.2, *P* = 0.6).

**Table 1:** Descriptive statistics and univariable logistic regression analysis of categorical variables investigated for association with *Cryptosporidium* spp. infection (*n*, number; CI, confidence interval; *β*, regression coefficient; SE, standard error; OR, odds ratio; *P*, Wald *p* value)

Variable	Level	*n*	Infected	% (95% CI)	*β*	SE (*β*)	OR (95% CI)	*P*
Sex	Female	473	125	26.4 (22.5–30.4)	Ref.			
	Male	134	49	36.6(28.4–44.7)	0.4	0.2	1.5 (0.96–2.4)	0.07
Strongyle infection	No	537	156	29.1 (25.2–32.9)	Ref.			
	Yes	70	18	25.7 (15.5–35.9)	− 0.3	0.3	0.97 (0.5–1.8)	0.9
*n* horses owned	One	369	109	29.5 (24.9–34.2)	Ref.			
	≥ 2	238	65	27.3 (21.7–33.0)	− 0.05	0.2	0.9 (0.6–1.4)	0.8

## Discussion

The current study provides evidence-based information on the prevalence of and potential risk factors for *Cryptosporidium* spp. infection in working horses in Egypt. The prevalence of *Cryptosporidium* spp. reported in the present study (28.7%) was higher than reported prevalences in farm animal species in Egypt such as cattle (10.2%), buffalo (0.5–12.3%), and sheep (1.3%) (Ibrahim et al. 2016; Mahfouz et al. 2014). Two studies on dogs from Egypt reported prevalences comparable to the current study (30–34%) (Awadallah and Salem 2015; Gharieb et al. 2018). These four studies conducted in Egypt also used the modified Zielh-Neelsen staining technique and light microscopy to identify *Cryptosporidium* spp. oocysts in faeces. All horses identified with positive *Cryptosporidium* spp. infection in the current study were clinically normal with no clinical signs or history of diarrhoea. This is consistent with previous studies in horses (Tuemmers et al. 2023; Xu et al. 2023) and indicates that infection has minimal clinical significance in immunocompetent adult horses.

Several studies have investigated the prevalence of *Cryptosporidium* spp. in foals and adult horses and have reported variable prevalence estimates. The reported prevalence estimates varied according to the geographic location of the population studied and the detection methods used. Studies from China that used polymerase chain reaction (PCR) to investigate *Cryptosporidium* spp. prevalence in adult horses reported an average prevalence of 1.8–3.1% (Li et al. 2019; Qi et al. 2015; Xu et al. 2023) which is much lower than the prevalence reported here. A recent systematic review and meta-analysis reported a pooled prevalence of 13.8% in studies that used microscopy compared with only 4.7% in studies that used PCR (Hatam-Nahavandi et al. 2019). Another recent study from Chile that investigated *Cryptosporidium* spp. infection prevalence in 100 adult horses using microscopy reported a higher prevalence of 67% (Tuemmers et al. 2023). Earlier studies on the prevalence of *Cryptosporidium* spp. infection in adult horses that used microscopy for identification of oocysts reported prevalences of 36.9% in Taiwan (Guo et al. 2014), 9.4% in Poland (Majewska et al. 1999), 18.4% in Brazil (Inacio et al. 2012), and 19.5% in Iran (Haghi et al. 2020).

Studies have consistently reported higher prevalence of *Cryptosporidium* spp. infection in foals than adult horses (Cole et al. 1998; Inacio et al. 2012; Tuemmers et al. 2023; Veronesi et al. 2010). Horse age was negatively associated with the prevalence of *Cryptosporidium* spp. infection in the present study, but this association was not statistically significant. The present study population included only 14 foals that were < 6 months old, half of which were found to be infected. The sex of the horse was also not associated with *Cryptosporidium* spp. infection in the current study which is consistent with previously published research (Tuemmers et al. 2023).

In the current study, we attempted to extract DNA from a random subset of positive samples and amplify 18S SSU rRNA genes using PCR; however, none of the PCR reactions yielded positive results. This could be attributed to low oocyst excretion by clinically normal adult horses (Kostopoulou et al. 2015) which may have resulted in a low DNA yield, especially if DNA was directly extracted from faeces without concentrating oocysts. Furthermore, the presence of PCR inhibitors in faecal samples could have impaired DNA amplification (Elwin et al. 2012). This was also consistent with prevalence studies of *Cryptosporidium* spp. infection which reported a lower prevalence when PCR was used as the sole detection method (Hatam-Nahavandi et al. 2019; Li et al. 2019; Qi et al. 2015; Xu et al. 2023). Overall, interpretation of epidemiological studies of *Cryptosporidium* spp. prevalence should consider the test characteristics (e.g., sensitivity and specificity) of the diagnostic methods used (Majewska et al. 2004). The detection threshold of the modified Ziehl–Neelsen staining technique has been estimated to be 10 × 10^5^ oocysts per gram of faeces (Cole et al. 1999; Weber et al. 1991). Furthermore, the presence of other acid-fast microorganisms in faeces, which are comparable in size to *Cryptosporidium* spp. oocysts, such as yeast, fungi, and other protozoa such as *Cyclospora*, could result in a low test specificity unless stained smears are examined by an expert technician (Nielsen and Ward 1999; Tahvildar-Biderouni and Salehi 2014). Therefore, the use of the modified Ziehl–Neelsen staining technique to diagnose *Cryptosporidium* ssp. infection may underestimate (high % of false negatives) or overestimate (high % of false positives) the prevalence, depending on the shedding intensity of oocysts and the level of experience of the diagnostician.

Previous studies in equine successfully genotyped *Cryptosporidium* spp. using the 18S SSU rRNA gene amplification and sequencing. *C. hominis* and *C. andersoni* were identified in horse faecal samples in China (Deng et al. 2017; Liu et al. 2015). Other recent studies in China reported that *C. parvum* and *C. hominis* were identified in faecal samples collected from racehorses and farmed donkeys (Wang et al. 2020; Xu et al. 2023). Findings from these studies suggest the potential zoonotic transmission of *Cryptosporidium* spp. between horses and human.

Another limitation of the current study was that samples were collected at a single time point; therefore, we did not consider intermittent shedding of *Cryptosporidium* spp. oocysts (Xu et al. 2023), which might have underestimated the reported prevalence in the current study. Furthermore, neither locations nor horses were randomly selected for inclusion in the current study which may limit the generalisability of our results. Notwithstanding these limitations, the current study provides evidence-based information regarding the prevalence of *Cryptosporidium* spp. infection in a population of working horses in Egypt. Further studies to genotype these parasites in this population of horses are required to investigate the zoonotic potential of the infection.

## Data Availability

The dataset generated during and/or analysed during the current study is publicly available at the figshare repository (https://figshare.com/s/8e171f9b5390eb21a2a2).
